# Pancreatic Cancers with High Grade Tumor Budding Exhibit Hallmarks of Diminished Anti-Tumor Immunity

**DOI:** 10.3390/cancers13051090

**Published:** 2021-03-04

**Authors:** Hassan Sadozai, Animesh Acharjee, Thomas Gruber, Beat Gloor, Eva Karamitopoulou

**Affiliations:** 1 Center for Sport, Exercise and Life Sciences, Coventry University, Coventry CV1 5FB, UK; hassan.a.sadozai@gmail.com; 2 Institute of Cancer and Genomic Sciences, Centre for Computational Biology, College of Medical and Dental Sciences, University of Birmingham, Birmingham B15 2TT, UK; a.acharjee@bham.ac.uk; 3 Institute of Translational Medicine, University Hospitals Birmingham NHS Foundation Trust, Birmingham B15 2TH, UK; 4 NIHR Surgical Reconstruction and Microbiology Research Centre, University Hospital Birmingham, Birmingham B15 2WB, UK; 5 Independent Scholar, NCIS—National Coalition of Independent Scholars, CH-3930 Visp, Switzerland; thomas.gruber@ncis.org; 6 Department of Visceral Surgery, Insel University Hospital, University of Bern, CH-3010 Bern, Switzerland; Beat.Gloor@insel.ch; 7 Pancreatic Cancer Research Group, Institute of Pathology, University of Bern, Murtenstrasse 31, CH-3008 Bern, Switzerland

**Keywords:** pancreatic ductal adenocarcinoma (PDAC), tumor budding, gene signature, T lymphocytes, T cell-enriched, M1/M2 macrophages

## Abstract

**Simple Summary:**

Pancreatic cancer, in its most common manifestation pancreatic ductal adenocarcinoma (PDAC), is a uniquely lethal disease with very limited treatment options and few prognostic biomarkers. Tumor budding is a proven independent, adverse prognostic factor in many tumor types including PDAC. Tumor buds can be detected histologically as single cancer cells or clusters of up to four cancer cells at the tumor invasive front. Tumor budding is biologically correlated to the induction of epithelial-mesenchymal transitions (EMT) and disease progression. In this study, we sought to investigate the immunological composition of tumors with high levels of tumor budding. We show that PDAC cases with a high grade of tumor budding display notably diminished anti-tumor immunity. These findings were further validated by gene expression analysis of PDAC cases from The Cancer Genome Atlas (TCGA). Our results provide insight on the immune escape mechanisms of tumor cells undergoing EMT. This offers the potential of designing novel treatments combining immunotherapies with EMT-targeted drugs.

**Abstract:**

Tumor budding is associated with epithelial-mesenchymal transition and diminished survival in a number of cancer types including pancreatic ductal adenocarcinoma (PDAC). In this study, we dissect the immune landscapes of patients with high grade versus low grade tumor budding to determine the features associated with immune escape and disease progression in pancreatic cancer. We performed immunohistochemistry-based quantification of tumor-infiltrating leukocytes and tumor bud assessment in a cohort of *n* = 111 PDAC patients in a tissue microarray (TMA) format. Patients were divided based on the ITBCC categories of tumor budding as Low Grade (LG: categories 1 and 2) and High Grade (HG: category 3). Tumor budding numbers and tumor budding grade demonstrated a significant association with diminished overall survival (OS). HG cases exhibit notably reduced densities of stromal (S) and intratumoral (IT) T cells. HG cases also display lower M1 macrophages (S) and increased M2 macrophages (IT). These findings were validated using gene expression data from TCGA. A published tumor budding gene signature demonstrated a significant association with diminished survival in PDAC patients in TCGA. Immune-related gene expression revealed an immunosuppressive TME in PDAC cases with high expression of the budding signature. Our findings highlight a number of immune features that permit an improved understanding of disease progression and EMT in pancreatic cancer.

## 1. Introduction

Pancreatic adenocarcinoma (PDAC) represents the most common form of pancreatic cancer and remains resistant to nearly all treatments [1]. As such, the five-year survival rate for PDAC patients remains below 10% and only surgical resection in combination with multi-agent chemotherapy offers a prospect for long-term survival [1,2]. Moreover, approximately 60% of patients present with advanced, metastatic disease and only between 10–20% of patients are eligible for surgical resection [2]. Mutations in four genes (*KRAS*, *TP53*, *SMAD4* and *CDKN2A*) have been identified to be the primary drivers of PDAC [1,3]. As in other tumor types, disease progression in PDAC is associated with tumor stage, lymph node metastases and involvement of the resection margins [4,5,6]. In recent years, a number of groups, including ourselves, have demonstrated that tumor budding is negatively correlated to overall survival (OS) in PDAC [7,8,9].

Tumor budding is denoted by de-differentiated tumor cells, observed as single cells or small clusters of up to four cells at the invasive front of many cancer types and is considered to be associated with epithelial-mesenchymal transition (EMT) [10]. EMT is purported to be among the first steps of a complex biological pathway which governs tumor progression and in a number of cancer types, including PDAC, tumor budding is found to be associated with distant metastasis [10,11]. To date, the systemic and intratumoral immunological mechanisms which promote metastasis are incompletely understood [12]. We previously characterized three distinct immune subtypes in PDAC, with over 50% of cases displaying an “immune-escape” phenotype poor in effector T cells but enriched in FOXP3^+^ Tregs and displaying high levels of tumor budding [13]. However, a comprehensive immune landscape of pancreatic cancers with high grade tumor budding has not yet been reported. Understanding the immune correlates of increased tumor budding and disease progression in PDAC, offers significant promise for devising combinatorial treatments.

In this study, we examined the immune contexture of PDAC cases with high grade versus low grade tumor budding. We show that cases with high grade tumor budding exhibit key hallmarks of an immunosuppressive tumor microenvironment (TME). Thus, high grade budding is associated with diminished dendritic cells (DC), T cells and immunostimulatory M1 macrophages. Finally, we utilized RNA-sequencing (RNA-seq) data from pancreatic cancer samples in The Cancer Genome Atlas (TCGA) to dissect the tumor immune landscape of cases with high expression of a tumor budding gene signature.

## 2. Results

### 2.1. Tumor Budding Grade Is a Negative Prognostic Factor in PDAC

**Figure 1:**
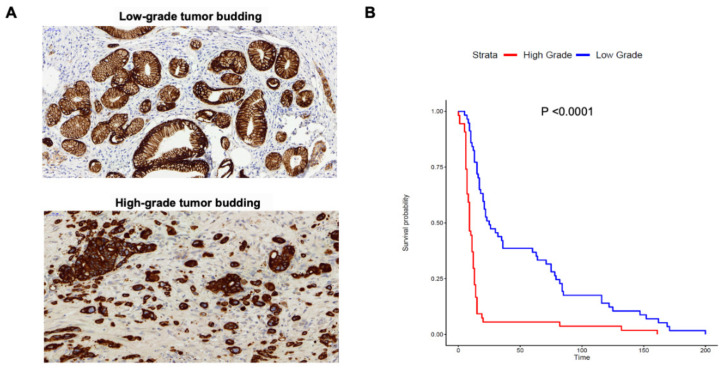
Tumor budding grade is associated with overall survival in PDAC. (**A**) Representative images of cases from High Grade vs. Low Grade tumor budding groups. (**B**) Kaplan-Meier curves comparing the OS of PDAC cases with High Grade (Category 3) versus Low Grade (Category 1&2) tumor budding. There were *n* = 54 cases in the High Grade versus *n* = 57 cases in the Low Grade budding group. Statistical comparisons were performed using the log-rank test.

The study design is shown in Supplementary Materials Figure S1. Based on previously published criteria, we categorized each PDAC patient as category 1 (0–4 buds), category 2 (5–9 buds) and category 3 (≥10 buds) based on the number of tumor buds detected [13,14]. Subsequently, patients were classified as High Grade (HG-Category 3) or Low Grade (LG-Categories 1 and 2 combined) as shown in Figure 1A. A comparison of clinical and pathological characteristics between HG and LG groups is shown in Table 1. No significant association was noted between tumor budding status and patient age, gender, tumor size, UICC stage and anatomic site. However, compared to LG cases, HG patients displayed significantly reduced OS and progression-free survival (PFS) and reduced presence of tertiary lymphoid tissue (TLT). Inversely, the HG group exhibited notably higher serum CA19-9 values, and a strong association with higher tumor grade. These results suggest that HG cases exhibit certain indicators of progressive disease. Using Kaplan-Meier analysis, we sought to visualize the association of tumor budding with survival. A significant positive association was observed between HG status and reduced OS (*p* < 0.0001) as shown in Figure 1B. Similarly, a strongly positive association was demonstrated between OS and higher tumor budding category or number of tumor buds (Figure S2A,B). Thus, our results confirm that tumor budding, is a strong prognostic indicator in PDAC, a finding that is supported by a recently concluded meta-analysis in PDAC patients [15]. Finally, the fibrotic profiles of cases with HG vs. LG budding were also assessed. However, no significant differences could be found for IHC staining with αSMA and Masson’s trichrome staining for collagen (Figure S3A,B). These results suggest that high levels of tumor budding are not associated with enhanced desmoplasia in pancreatic cancer.

### 2.2. Dissecting the Immune Landscape of High Grade versus Low Grade Budding Cases

Studies in other tumor types, in particular, colorectal cancer have shown the importance of both adaptive and innate immune cells (i.e., macrophages) in restricting tumor budding [10]. However, to date, the immune contexture of pancreatic cancer cases with high grade budding has not been well-defined. As such, immunohistochemistry (IHC) was utilized to detect and quantify the density (cells/mm^2^) of both adaptive and innate immune cells in the TME. We enumerated total T cells (CD3), cytotoxic T cells (CD8), helper T cells (CD4), Tregs (FOXP3), B cells (CD20), total macrophages (CD68), M1 macrophages (iNOS), M2 macrophages (CD163) and mature DC (DC-LAMP). Immune cell densities were evaluated independently in the intratumoral (IT), and stromal (S) compartments. Representative images of IHC staining for the aforementioned immune cell subsets are shown in Figure S4.

**Figure 2:**
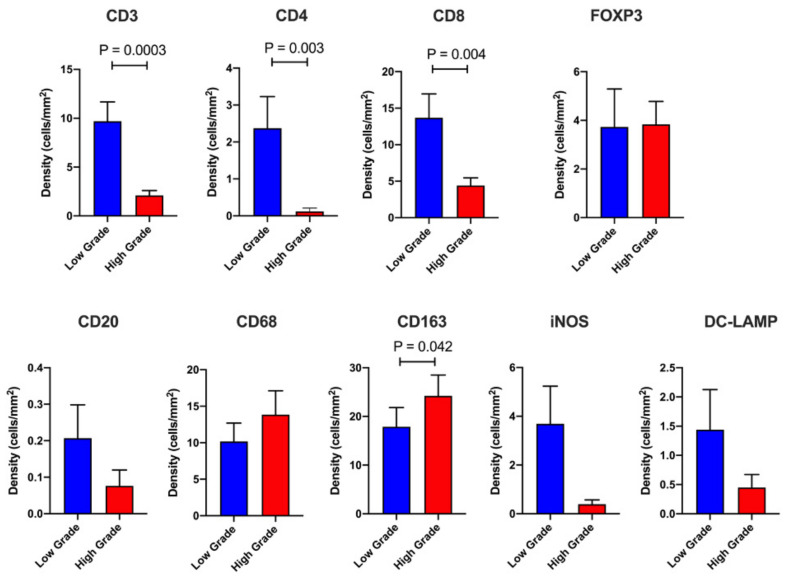
Intratumoral (IT) immune profiling. Barplots displaying the IT leukocyte density in High Grade (*n* = 54) versus Low Grade (*n* = 57) tumor budding cases. The following immune cell markers were utilized (CD3, CD4, CD8, FOXP3, CD20, CD68, iNOS, CD163, DC-LAMP). Data are depicted as mean + standard error (SE). Differences between groups were analyzed using the Mann-Whitney *U* test. Only significant *p*-values (<0.05) are shown on the graph.

It is pertinent to note that a majority of cases displayed few or no immune cells within the IT compartment. However, when compared to the LG group, patients in the HG cohort show marked differences in their IT immune contexture (Figure 2 and Table S1). Compared to LG patients, cases with HG tumor budding exhibit significantly lower densities of CD3^+^, CD4^+^ and CD8^+^ T cells (*p* = 0.0003, 0.003 and 0.004 respectively). There was a trend towards higher total macrophages (CD68^+^) lower B cells, M1 macrophages and DC in the HG group compared to LG budding cases, but these values did not achieve significance due to a large number of cases with no IT infiltration for these cell types. However, HG patients displayed elevated IT CD163^+^ M2 macrophages. These results suggest that a T cell-inflamed IT compartment contributes to restraining tumor budding in PDAC.

**Figure 3:**
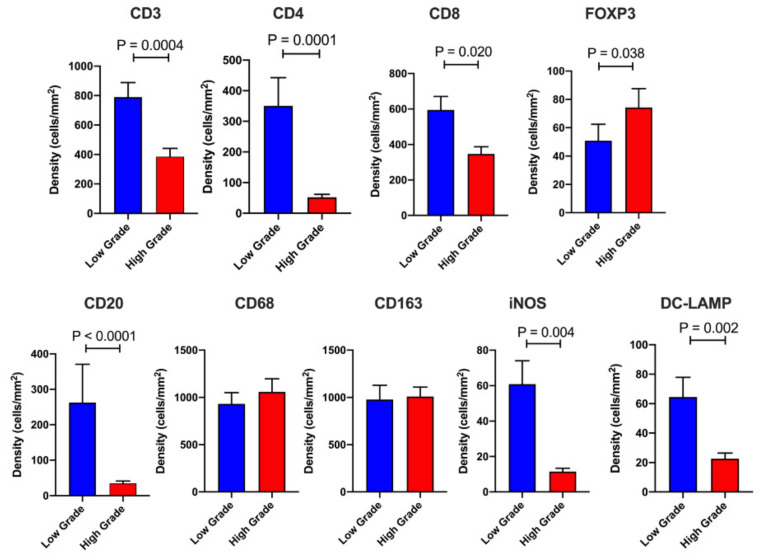
Stromal (S) immune profiling. Barplots displaying the S leukocyte density in High Grade (*n* = 54) versus Low Grade (*n* = 57) tumor budding cases. The following immune cell markers were utilized (CD3, CD4, CD8, FOXP3, CD20, CD68, iNOS, CD163, DC-LAMP). Data are depicted as mean + SE. Differences between groups were analyzed using the Mann-Whitney *U* test. Only significant *P* values are shown on the graph.

The overall densities of stromal leukocytes were many fold higher than in the IT compartment. However, the immune profile of HG versus LG cases was distinctly immunosuppressive (Figure 3 and Table S2). Compared to the LG group, the HG cohort displayed significantly fewer CD3^+^, CD4^+^ and CD8^+^ T cells (*p* = 0.0004, 0.0001 and 0.020 respectively). Inversely, FOXP3^+^ Tregs were found to be elevated in HG budding patients (*p* = 0.038). Cases with HG tumor budding also exhibited additional features suggesting inhibition of anti-tumor immunity. Compared to LG patients, HG cases display markedly lower densities of CD20^+^ B cells (*p* < 0.0001), M1 (iNOS^+^) macrophages (*p* = 0.004) and mature DC (*p* = 0.002). No significant differences were observed between HG and LG groups for the densities of total macrophages (CD68^+^) or M2 (CD163^+^) macrophages. These findings suggest that both innate and adaptive immune cells (such as M1 macrophages) prevent tumor budding and disease progression in pancreatic cancer.

Collectively, our results suggest that anti-tumor immunity is markedly diminished in cases with HG budding, thereby limiting immune-mediated disease control. TLT or tertiary lymphoid structures (TLS) are ectopic lymphoid aggregates that form in inflamed tissues including in cancer [16]. The presence of TLT is associated with improved survival in a number of tumor types including PDAC [17]. To determine the association between TLT status and budding grade, we assessed the formation of TLT in PDAC cases (Figure S5A). A strong association was observed between the presence of TLT and LG budding status as shown in Figure S5B. The proportion of HG cases with TLT (4%) was significantly lower (*p* < 0.0001) than LG cases with TLT (42%). We also performed principal component analysis (PCA), a dimensionality reduction technique, on IT and S leukocyte densities (Figure S6A,B). The results from the PCA demonstrated distinct clustering of HG vs LG cases based on their IT and S immune profiles. These data indicate that HG and LG budding cases have immunologically distinct TMEs and warrant personalized approaches to treatment. Finally, multivariate Cox regression was performed on IT and S leukocyte counts as well as other clinical parameters including tumor budding to determine which variables could independently predict OS (Table 2). The results showed that stromal CD4^+^, CD8^+^ and FOXP3^+^ densities and CD8^+^ T cells and intratumoral CD163^+^ macrophages are independent prognostic variables in our cohort. UICC stage IV and tumor budding number were also observed to be independent prognostic factors.

### 2.3. Prognostic Significance of a Tumor Budding Gene Signature

**Figure 4:**
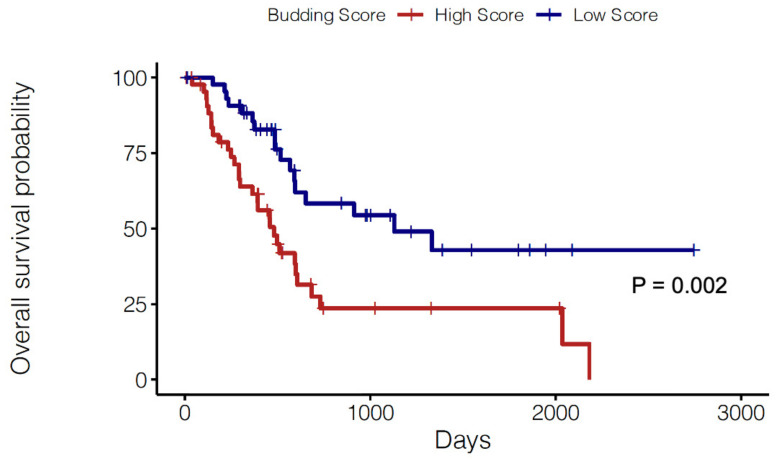
Prognostic role of tumor budding gene signature. Kaplan-Meier curves comparing the OS of PDAC cases from TCGA with a High (red) vs. Low (blue) Score for the molecular budding signature. Cases were dichotomized as high or low by top and bottom quartiles of expression (*n* = 45 patients each). Statistical comparisons were performed using the log-rank test. The budding gene score was computed using the *singscore* package in Bioconductor (see methods).

In order to validate our observations, we performed computational analyses of RNA-seq data from the PAAD (i.e., PDAC) cohort in TCGA. To determine which cases had higher levels of tumor budding, we scored PDAC samples from TCGA for the expression of a recently reported tumor budding gene signature [18]. This was performed using *singscore*, a rank-based gene expression scoring method [19]. Subsequently, patients were divided into a High Score (HS) and Low Score (LS) group based on quartile dichotomization (Q1 vs. Q4) of tumor budding gene score values (*n* = 45 patients each). When HS and LS cases were compared using Kaplan-Meier analysis (Figure 4), it was observed that HS cases displayed significantly reduced OS (*p* = 0.002). Thus, these observations provide a strong rationale for the prognostic significance of tumor budding in PDAC.

### 2.4. In Silico Immune Profiling of High Score versus Low Score Budding Cases

Next, we sought to dissect the immune features of TCGA cases with high expression of the tumor budding signature. As such, the proportion of both immune and non-immune cell types in HS and LS cases were estimated using the Estimate the Proportion of Immune and Cancer cells (EPIC) algorithm [20]. Cell-type deconvolution algorithms such as EPIC rely on reference gene expression profiles for specific cell types to predict their abundance in bulk tumor transcriptional datasets (RNA-seq or microarray) [21]. The EPIC algorithm was chosen so that the proportions of other cell types such as endothelial cells and cancer-associated fibroblasts (CAFs) in HS vs. LS patients could also be computed, thereby yielding a comprehensive assessment of the TME.

**Figure 5:**
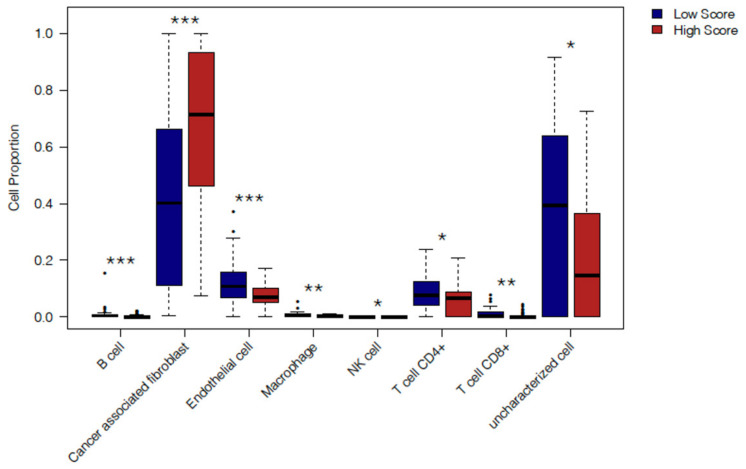
Cell type deconvolution of TCGA PDAC cases with High and Low Budding gene signature scores. Boxplots comparing the cell fractions as computed by the EPIC algorithm for immune and non-immune cells (B cells, CAFs, Endothelial cells, Macrophages, NK cells, CD4^+^ T cells, CD8^+^ T cells and uncharacterized cells) in High vs. Low Budding score cases (*n* = 45 patients each) from TCGA. Data are shown as box and whisker plots with the box representing the IQR and the whiskers extending to the minimum and maximum values. Statistical comparisons between High and Low Score cases were performed for each immune cell subset individually using the Mann-Whitney *U* test. Only significant *p* values (<0.05) are shown on the graph. *** *p* < 0.001, ** *p* < 0.01, * *p* < 0.05.

HS cases were found to exhibit distinct differences in the proportions of immune and non-immune cells in their TME compared to the LS group (Figure 5 and Table S3). HS cases displayed a higher cellular fraction of CAFs and lower proportions of endothelial cells relative to LS patients (*p* = 0.0001 and 0.0004, respectively). The EPIC algorithm also computes the values of “uncharacterized cells”, which do not fit the reference profiles for the other cell types included in the algorithm (e.g., cancer cells or normal epithelial tissue) [20]. HS cases exhibited a lower cellular fraction of “uncharacterized cells” compared to LS patients (*p* = 0.02). Both CD4^+^ and CD8^+^ T cell quantities were estimated to be lower in HS cases vs. LS cases (*p* = 0.02 and *p* = 0.006 respectively). The HS group also exhibited significantly lower fractions of B cells (*p* = 0.0005), macrophages (*p* = 0.004) and higher proportions of NK cells (*p* = 0.03) compared to the LS group. Taken together, these findings suggest that immunological differences between HS and LS groups are concomitant with alterations in non-immune components of the TME.

**Figure 6:**
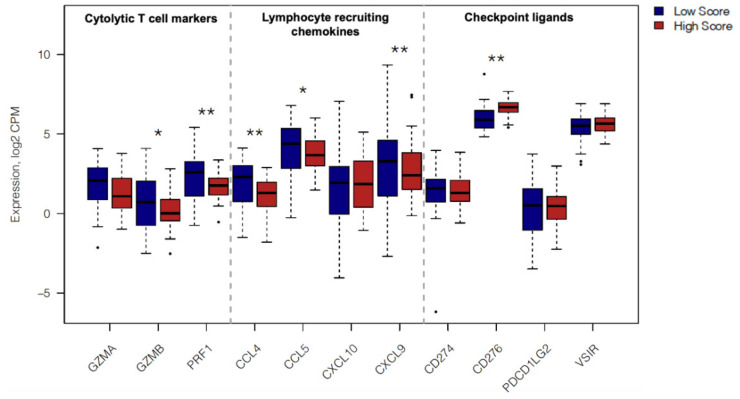
Immune related gene expression in TCGA PDAC cases with High and Low Budding gene signature scores. Boxplots comparing gene expression of immunological genes in High and Low Budding Score patients from TCGA (*n* = 45 patients each). Gene expression is shown as log2-transformed, normalized CPM (counts per million) values. Transcriptome-wide differential expression statistics were computed using EdgeR under quasi-likelihood framework. P values were corrected for multiple testing using the Benjamini-Hochberg method limiting the correction to the 11 genes of interest displayed above. Only significant *p* values (<0.05) are shown on the graph. ** *p* < 0.01, * *p* < 0.05.

Both HG patients in situ and HS samples from TCGA exhibited markedly reduced lymphocyte infiltration. As such, we performed differential gene expression (DEG) profiling (Figure 6) between HS and LS PDAC patients for a number of key genes associated with T cell-inflamed tumors [22,23]. First, we assessed for the expression of the following genes associated with cytotoxic cells (T cells and NK cells); granzyme A (*GZMA*), granzyme B (*GZMB*) and perforin (*PRF1*). HS cases exhibited significantly lower expression for *GZMB* and *PRF1* (*p* = 0.04 and 0.003, respectively). Second, we examined gene expression levels for a selection of chemokines associated with T cell recruitment to the tumor, namely *CCL4, CCL5, CXCL9, CXCL10* [22]. It was observed that compared to LS patients, HS PDAC tumors show significantly lower levels of *CCL5* and *CXCL9* (*p* = 0.02 and 0.006, respectively). Finally, we assessed the gene expression of a selection of checkpoint ligands; *PD-L1* (*CD274*), *PD-L2* (*PDCD1LG2*), *B7-H3* (*CD276*) and *VISTA* (*VSIR*) [24]. Compared to LS patients, the HS group displayed no differences in expression values for any of the aforementioned checkpoint ligand genes apart from B7-H3, which was found to be significantly higher in HS cases (*p* = 0.003). Thus, our in silico results show that multiple features of diminished T cell activation and reduced lymphocyte infiltration are associated with enhanced tumor budding in PDAC.

## 3. Discussion

Currently, only a small number of treatment options are available for pancreatic cancer and even in resectable PDAC, the five year survival rate is only approximately 30% [25]. A systematic review of the literature has shown that tumor budding is an independent, prognostic biomarker in PDAC [15]. However, therapy selection and patient stratification on the basis of tumor budding will require an understanding of the mechanisms that permit EMT and disease progression. Tumor immunotherapy has emerged as a key pillar of cancer treatment, and while no immunotherapies have received regulatory approval for first-line treatment of PDAC, a number of approaches show promise in pre-clinical and clinical studies [25,26]. In this report, we show that pancreatic cancers with high levels of tumor budding exhibit distinct features of an immunosuppressive TME. Thus, integrating tumor budding profiles with analyses of the immune contexture can offer significant opportunities for personalizing cancer immunotherapies for pancreatic cancer.

A significant body of evidence has shown that tumor budding in cancers is associated with EMT [10,27]. Compared to the bulk of the tumor, tumor budding cells in PDAC are reported to express increased levels of the EMT-related transcription factors ZEB1 and ZEB2 and decreased expression of the cell-to-cell adhesion protein, E-cadherin [28]. Canonically, the EMT process is associated with hallmarks of cancer progression such as tumor stemness, tumor cell migration, metastasis and resistance to treatment [29]. In a murine model of autochthonous PDAC, it was shown that abrogation of EMT did not alter cancer cell invasion or metastasis but significantly increased tumor cell sensitivity to gemcitabine [30]. In the meta-analysis of the PDAC literature by Lawlor et al., it was observed that there were no significant differences in terms of lymph node metastasis between patients with or without high grade tumor budding [15]. Thus, the EMT process in PDAC ostensibly fosters disease progression through improving tumor cell survival and resistance to treatment. Due to the important role of EMT in fostering disease progression, a number of EMT-targeted drugs are in preclinical and clinical development such as STAT3 inhibitors (e.g. Napabucasin) or metabolic inhibitors such as Simvastatin [31,32]. However, in order to achieve optimal outcomes with anti-EMT drugs, a more comprehensive understanding of the TME of HG budding cases is required.

The interaction between tumor cells undergoing EMT and the immune landscape is a dynamic process, as EMT has been shown to result in immunosuppression, and inflammatory immune signals can induce EMT in carcinoma cells [33,34]. However, as per the concept of cancer immunoediting, tumor cells are continually targeted by immune cells but eventually evade immune destruction and progress to metastatic disease [35]. In our study, we noted that HG tumor budding is associated with multiple features of immune escape. Compared to LG budding cases, HG patients exhibited markedly diminished CD4^+^ T helper cells and CD8^+^ cytotoxic T cells in both the IT and S compartment. Moreover, cases with HG tumor budding also displayed reduced numbers of stromal B cells and mature DC concomitantly with increased numbers of stromal FOXP3^+^ Tregs. These findings are in accordance with the observation that only 4% of HG budding cases displayed TLT/TLS formation. TLS are composed of DC-LAMP^+^ mature DC and B cells, and putatively serve as regions for T cell priming and activation [36]. Finally, cell-type deconvolution and signature-based scoring revealed that PDAC cases from TCGA with high levels of the budding gene signature, recapitulated an “immune-escape” phenotype with diminished T cells and B cells. Our results are supported by the work of Fujiyoshi et al., who utilized multiplex immunofluorescence to show that tumor budding numbers were inversely associated with CD3^+^CD8^+^ cytotoxic T cells [37]. Conversely, in hepatocellular carcinoma (HCC), patients with HG tumor budding were shown to have higher densities of CD8^+^ T cells and CD20^+^ B cells [38]. These seemingly contrary observations can be explained by the “attacker-defender model”, where tumor budding represents an aggressive disease phenotype which elicits an enhanced immune response [10]. These observations also fall within the paradigm of the cancer immunoediting concept, as tumors are constantly evolving to escape immune detection and destruction [39].

Our data demonstrates that EMT in PDAC, occurs in the context of an immune evasive tumor microenvironment. There is tremendous evidence from the literature showing that EMT induces multiple mechanisms of immune evasion [34]. Previous studies have shown that carcinoma cells undergoing EMT exhibit increased resistance to cell-mediated cytotoxicity from both CD8^+^ T cells and NK cells [40,41]. Furthermore, Mak et al. reported an EMT gene score derived from patients with multiple cancer types, excluding PDAC, and showed that high EMT scores were associated with increased expression of checkpoint ligands such as PD-L1, PD-L2 and B7-H3 (CD276) in various solid organ malignancies [42]. Our report provides further proof for this. By analyzing the transcriptional profiles of PDAC patients from TCGA we demonstrate that higher gene expression of the tumor budding gene signature was correlated to lower expression of cytolytic T cell markers and lymphocyte recruiting chemokine genes. In particular, the chemokine genes *CCL4, CCL5* and *CXCL9*, which were found to be diminished in HS versus LS cases from TCGA, were previously reported to be part of a gene signature associated with enhanced T cell infiltration in melanoma [43]. We also observed increased gene expression of the checkpoint ligand *B7-H3* (*CD276*) in HS patients but not that of *PD-L1 (CD274)* or *PD-L2 (PDCD1LG2)*. While B7-H3 has been shown to have non-immunological functions in promoting tumor cell migration and invasion, it is also currently recognized as a potent inhibitor of T cell activation, although its receptor remains unknown [44,45]. Enoblituzumab (MGA271), a monoclonal antibody targeting B7-H3, is currently in clinical trials and antibody-drug conjugates targeted to B7-H3 are being explored in a number of cancer types [45]. Therefore, HG tumor budding might serve as a biomarker for selecting PDAC cases that can benefit from Enoblituzumab immunotherapy, although this requires validation.

While T cells play a crucial role in controlling tumor progression, the TME is also shaped by innate immune cells particularly tumor associated macrophages (TAMs) and DC [46,47]. Immunosuppressive TAMs can inhibit anti-tumor immunity through direct and indirect mechanisms. These include but are not limited to, inhibiting DC activation, direct suppression of T cells through checkpoint ligand expression and modulation of the vasculature [47,48] Conversely, putative M1 or immunostimulatory macrophages can induce DC maturation, T cell recruitment and display tumor cell killing [48]. In PDAC cases with HG versus LG tumor budding, we noted intriguing differences between the density of M1 and M2 macrophages. In the IT compartment, M2 macrophages were significantly elevated in HG patients and M1 macrophage densities tended to be reduced, but these differences did not achieve statistical significance. However, in the stroma, HG budding cases displayed markedly diminished presence of M1 macrophages compared to LG budding cases with no differences in the levels of M2 macrophages between both groups. Given that CD68 (macrosialin) is a pan-macrophage marker, it can be expected that differences in total CD68^+^ macrophage counts would not achieve significant differences between budding groups given the higher density of M2 and lower density of M1 macrophages in HG cases compared to LG cases. It is pertinent to note that CD68^+^ can also be expressed in other myeloid cell subsets including granulocytes [49]. In the scientific literature, it has been shown that while M2 macrophages are capable of inducing EMT through a number of pathways [50,51], EMT in both benign and cancerous pancreatic cell lines can be induced by M1 and M2 macrophage subsets [52]. Indeed, TAMs have been shown to influence nearly all aspects of tumor progression from angiogenesis to invasion and metastasis [53]. Furthermore, while M1 and M2 macrophages offer representative phenotypes for TAMs with respectively pro- and anti-tumor capabilities, TAMs in situ display remarkable heterogeneity in phenotype and function [53]. Cell-type deconvolution of HS versus LS cases in TCGA also showed that LS cases exhibited significantly higher macrophages. However, given that the EPIC algorithm does not compute estimates of macrophage subsets, and provided that reference-profile based cell deconvolution methods are currently still at an early stage of development, the precise identities of the higher macrophage proportions in LS cases cannot be further dissected [21]. Nevertheless, our results demonstrate, at least in part, a potent role for M1 macrophages in shaping the immunostimulatory TME observed in cases with LG tumor budding. In a recent report by Garrido-Martin et al., the authors showed through single-cell transcriptomics in lung cancer, that while a majority of TAMs exhibited M2-like properties, 25% of cases exhibited macrophages with M1-like features, which were implicated in recruiting T cells via CXCL9 [54]. In our study, LS tumor budding cases from TCGA demonstrated significantly higher *CXCL9* expression compared to HS patients suggesting that M1 macrophages are a key cellular source of CXCL9 in PDAC.

M1 macrophages have also been shown to function as lymphoid tissue-inducer (LTi) cells, fostering TLS development in murine models of atherosclerosis, thereby suggesting their potential for inducing TLS formation in the context of cancer [55]. The markedly lower proportion of HG budding patients with TLS presence and concomitantly diminished M1 densities offers support to the notion of M1 macrophages acting as LTi cells in PDAC. These results offer a strong rationale for therapies that repolarize TAMs to an M1-like phenotype, such as anti-CD40 agonist antibodies, for tackling treatment-refractory PDAC cases with HG budding and highly immunosuppressed TMEs [53]. Recent evidence for this approach was shown in a seminal study by Panni et al. who targeted the surface integrin CD11b/CD18 through a molecular agonist (ADH-503) in murine PDAC models [56]. ADH-503 was shown to have a broad-spectrum role in repolarizing TAMs, improving T cell responses and enabling responses to anti-PD-1 checkpoint therapy in non-responsive PDAC mouse models [56]. Thus, tumor budding might serve as a useful biomarker to highlight PDAC cases which might benefit the most from myeloid-cell repolarization therapies that are currently in preclinical and clinical development.

Our data also showed that there was a trend towards increased intratumoral DC and significantly higher densities of stromal DC in LG cases compared to HG patients. Similar to TAMs, distinct DC subpopulations can enhance or suppress anti-tumor immune responses [46]. In recent years, an increasing body of evidence has highlighted the crucial roles of a subset of conventional DC (cDC), termed cDC1 in orchestrating the immune response to cancer [46]. Despite being present in tumors at low frequencies, these Batf3-dependent cDC1 are critical for intratumoral T cell priming and recruiting T cells via chemokines such as CXCL9 and CXCL10 [57,58]. Therefore, it is possible that cDC1 contribute to the higher gene expression of *CXCL9* observed in PDAC cases with Low budding gene signature scores. Conversely, other DC subsets such as plasmacytoid DC (pDC) can promote immune tolerance and produce multiple immunoregulatory molecules such as IL-10 and TGFβ [46]. The diversity of DC functions in the TME was demonstrated in a recent report [59]. In this study, the authors found that metastatic lymph node positive status was associated with M2 and activated DC profiles in silico. The authors also provided evidence that WNT pathway signaling promotes an immunosuppressive milieu in PDAC, particularly via pancreatic tumor cell interactions with DC [59]. This study shows that DC phenotypes and functions in PDAC remain poorly described. Despite the fact that DC are found in low densities in the TME, they have a marked influence on disease progression [46]. As such, further study using multiplex immunophenotyping approaches are required to unveil the complexity of the DC landscape in PDAC.

Finally, in silico cell-type deconvolution revealed important differences between TCGA cases with high expression versus low expression (i.e., HS vs. LS) of tumor budding gene scores in terms of their non-immunological cell landscape. CAFs, which are implicated in promoting EMT and tumor budding, have also been recognized for their capacity to suppress both adaptive and innate immunity [10,60]. HS cases displayed significantly higher proportions of CAFs, as computed by EPIC. Furthermore, HS cases showed significantly lower abundance of endothelial cells, potentially representing an aberrant tumor vasculature as well as lower proportions of “uncharacterized cells”. As described in the original report describing the EPIC algorithm, these cells can represent malignant cells, but also normal epithelial tissue [20]. The diminished expression of uncharacterized cell signature in HS cases might signal a transcriptional shift from epithelial carcinoma cells, to a mesenchymal phenotype. It has been shown in colorectal cancer that micro-dissected tumor budding regions exhibit notable transcriptional differences compared to the tumor bulk [61]. Thus, while our in silico gene expression profiling provides some evidence on the composition of non-immune components of the TME in HG budding cases, these require further validation at the protein level in pancreatic tumor tissue.

Our study is limited by a small sample size and we did not have access to a secondary tissue cohort for validation purposes. Therefore, we sought to recapitulate our findings using RNAseq data from TCGA. However, immune cell deconvolution techniques such as EPIC can predict the relative abundance of leukocytes in tumor tissue but cannot replace profiling performed through immunohistochemistry or flow cytometry [21] As such, further our work requires validation in PDAC tumor tissue. High dimensional phenotyping technologies such as CyTOF and single-cell RNA-seq will be required to accurately dissect the immunological heterogeneity of the TME in pancreatic cancers with high grade tumor budding [62,63]. Nonetheless, our study provides a multifaceted view of the immune landscape of pancreatic cancers with high grade tumor budding and highlights the need for targeting EMT in order to improve immunotherapy outcomes in PDAC.

## 4. Materials and Methods

### 4.1. Patient Cohort

The study design is depicted in Figure S1. The cohort for this study consisted of a total of 111 surgically resected PDACs (stage I–III) in ngTMA^®^ format (see below). The study population included a sub-cohort of 25 long-term progression-free and overall survivors (OS ≥ 60 months). As such, the number of evaluated cases in each group are provided in the figure legend. Patients were selected on the base of tissue availability and accessibility to full follow-up information and overall survival (OS). All patients provided written informed consent for inclusion in this study and the study was approved by the Ethics Committee of the canton Bern (KEK Nr 200/14).

### 4.2. Next-Generation Tissue Microarray (ngTMA) Construction

Tissue microarrays were constructed using the ngTMA^®^ approach [64]. For each patient, one hematoxylin and eosin (H&E) stained representative whole tissue slide was scanned (Panoramic P250, 3DHistech, Budapest, Hungary). Using a tissue microarray annotation tool of 0.6 mm in diameter, slides were digitally annotated and punches were obtained from 8 different tumor regions to account for tumor heterogeneity. Next, corresponding formalin-fixed (10% buffered formalin) paraffin-embedded tissue blocks were loaded into an automated tissue microarrayer (TMA Grandmaster, 3DHistech, Budapest, Hungary). The digital slides were aligned with the corresponding donor block. Annotated regions were cored from the donor block and transferred to the recipient ngTMA^®^.

### 4.3. Assessment of Tumor Budding and TLT Status

Tumor budding has been previously described and was defined as single tumor cells or tumor cell clusters of up to four cells [14]. Whole tissue sections of the PDACs, stained with H&E as in routine diagnostics, were utilized. Briefly, one hotspot with the highest density of tumor buds was selected at low magnification. Then, tumor buds were counted at 20× magnification (area 950 μm^2^). The number of tumor buds counted in that area was divided by 1.21 to obtain the number of buds in an area of 785 μm^2^ according to the ITBCC method [65]. Density of tumor buds was assigned into three categories: low budding (budding category (1): 0–4 buds; intermediate budding (budding category (2): 5–9 buds; and high budding (budding category (3): ≥10 buds. Patients were subsequently grouped into two budding grades as Low Grade (budding categories 1 and 2) and High Grade (budding category 3) for subsequent analyses. Cases were evaluated as present or absent for TLS/TLT based on detection in H&E-stained slides.

### 4.4. Immunohistochemistry

ngTMAs were sectioned at 3 μm, dewaxed and rehydrated in distilled H_2_O. They were double stained immunohistochemically for pancytokeratin (1:400, cytokeratin LMW, clone AE1/AE3, M3515, Dako-Agilent, Santa Clara, CA, USA) and each of the following: CD3 (1:400, clone SP7, ab16669, Abcam, Cambridge, UK), CD4 (1:100, clone CD4/4B12, M7310, Dako), CD8 (1:100, clone C8/144B, M7103, Dako), CD20 (1:100, clone L26, M0755, Dako), CD68 (1:100, clone KP1, M0814, Dako), DC-LAMP (1:100, CD208/DC-LAMP PA, 10527-H08H, Sino Biological, Beijing, China), iNOS (1:100, PAb, PA3-030A, Thermo Fisher Scientific, Waltham, MA, USA), CD163 (1:100, clone 10D6, NCL-CD163, Leica Biosystems AG, Muttenz, Switzerland) and FOXP3 (1:100, clone 236A/E7, ab20034, Abcam). Antigen retrieval was performed with Tris-HCl, pH 9 for 30 min at 95 °C. Antibody testing and staining protocols have been established and staining was performed by an automated Leica Bond RX System (Leica Bond RX, Leica Biosystems, Muttenz, Switzerland) with the Bond Polymer Refine Kit (with DAB as chromogen) and Bond Polymer Refine Red Detection Kit for the double staining (Leica Biosystems, Newcastle, UK).

### 4.5. Immune Cell Scoring and Normalization

All slides were scanned with their corresponding H&E-stained slides (Aperio Image Scope, Version 12.4.0.5043) and assessed by digital microscopy using the Case Viewer software (Case Viewer 3DHISTECH_Ltd Version 2.2.0.85100, 3DHistech, Budapest, Hungary). Immune cells in the intratumoral (IT) and stromal (S) compartments were enumerated separately and normalized per unit area as cells/mm^2^. For each immune cell population, the average counts across all TMA cores (5–8 cores) of the same patient were used for further analysis. Evaluation was performed by a pathologist blinded to clinical parameters. Intratumoral TILs are defined as lymphocytes in direct cell-to-cell contact with the tumor cells with no intervening stroma. Stromal TILs are located scattered or clustered in the stroma between the carcinoma cells/clusters and do not directly interact with tumor cells [66].

### 4.6. TCGA Data Acquisition and Analysis

RNA-seq data (counts) for 182 pancreatic cancer samples were downloaded from GDC portal (https://portal.gdc.cancer.gov/, accessed on 7 July 2020). Dataset was limited to primary tumors only. Full expression matrix was filtered to remove low-abundance genes (defined as gene with sample-average log2 CPM below median of all genes). Genes included in budding signature and immune marker genes of interest were retained even if they fell into low-abundance bucket. TMM normalization was applied to the filtered dataset to estimate normalization factors. These were used to compute log2-transformed CPM values (using prior count of 1), which, in turn, were utilized to compute the tumor budding signature. The molecular budding signature (i.e., tumor budding signature) was previously reported [18]. The 7 genes comprising this signature are *MSLN*, *SLC4A11*, *WNT11*, *SCEL*, *RUNX2*, *MGAT3* and *FOXC1*. The normalized signature scores for a given gene signature were calculated for each sample using the *singscore* package in BioConductor [19], following established user guidelines. Out of 177 samples which were available for analysis, the top and bottom quartile of tumor budding score cases were labeled “High Score” and “Low Score” respectively (*n* = 45 samples each).

In order to assess differential expression between High and Low Budding Score patients for genes of interest, the expression dataset described above was analyzed using Quasi-Likelihood framework in EdgeR [67]. Raw differential expression *p* values were corrected for multiple testing using the Benjamini-Hochberg method, limiting the correction to the 11 genes of interest. Expression distributions of genes of interest between High and Low Budding score patients were visualized as box plots using normalized Log2 CPM values.

### 4.7. Cell-Type Deconvolution

The EPIC algorithm is an approach to assess the abundance of 8 immune and non-immune cell types in bulk tumor RNA-seq profiles [20]. The EPIC algorithm was performed on High and Low Budding Score cases from the TCGA PAAD cohort using normalized unlogged CPM values as input and utilizing the ‘immunedeconv’ package in R, a uniform access-tool for multiple cell-type deconvolution methods [68]. Statistical significance of EPIC score differences between High and Low Budding Score pancreatic cancer samples were assessed using Mann-Whitney *U* Test.

### 4.8. Survival Analysis

We generated Kaplan-Meier plots and performed log-rank tests to determine differences in overall survival between High Grade and Low Grade budding groups in our cohort and between High and Low Budding Score patients from the TCGA cohort. These were plotted and analyzed in the *survminer* package in R [69]. The cut-offs for patient stratification for each survival curve are provided in the figure legends. Multivariate Cox Regression was performed using *survival* package in R accounting for patient sex, tumor stage and grade.

### 4.9. Statistical Analysis

Statistical differences between continuous variables or immune cell counts were analyzed using Mann-Whitney *U* test while differences between categorical variables were calculated by means of Fisher’s exact test or Chi-square test. Statistical analyses were conducted in Prism (Version 8.3, GraphPad Software, San Diego, CA, USA) and R (Version 4.0.0). All tests were two sided and *P* values were considered significant at (*p* < 0.05).

## 5. Conclusions

To date, the immune contexture of PDAC remains poorly understood. High grade tumor budding occurs in a TME that displays diminished adaptive and innate anti-tumor immunity. Our findings suggest an essential role for both CD4^+^ T cells and M1 macrophages in preventing this process and controlling disease progression. However, further studies in larger cohorts are essential to validate our findings. However, our results provide evidence for the “immune-escape” mechanisms that can be targeted to control disease progression and immunotherapy resistance in PDAC.

## Figures and Tables

**Table 1:** Comparison of clinical and pathological parameters between High and Low grade budding patients. Quantitative data are presented as group medians with interquartile range (IQR) in brackets. Categorical data are shown number of cases and as a percentage of survival subgroups in brackets.

Characteristic	High Grade, *n* = 54	Low Grade, *n* = 57	*p*-Value
OS (months)	9 (6–13)	25 (15–79)	**<0.001**
PFS (months)	5 (4–7)	18 (8–78)	**<0.001**
Sex			0.5
*F*	25 (46%)	31 (54%)	
*M*	29 (54%)	26 (46%)	
Age	64 (56–71)	67 (61–75)	0.053
Size (mm)	30 (25–40)	30 (25–35)	0.6
CA19-9 (U/mL)	687 (220–2123)	159 (95.5–597)	**0.0005**
Anatomic Site			0.33
*Head*	42 (78%)	49 (86%)	
*Others (Tail/Body)*	12 (22%)	8 (14%)	
Tertiary lymphoid tissue (TLT)			**<0.0001**
*Present*	2 (4%)	24 (42%)	
*Absent*	52 (96%)	33 (58%)	
Grade			**0.013**
*1*	6 (11%)	19 (33%)	
*2*	23 (43%)	22 (39%)	
*3*	25 (46%)	16 (28%)	
UICC Stage 8th Edition			0.065
*IA*	2 (3.7%)	3 (5.3%)	
*IB*	4 (7.4%)	13 (23%)	
*IIA*	2 (3.7%)	3 (5.3%)	
*IIB*	27 (50%)	30 (53%)	
*III*	16 (30%)	7 (12%)	
*IV*	3 (5.6%)	1 (1.8%)	

Bolded values denote significant results.

**Table 2:** Multivariate Cox regression analysis with model performance is summarized.

Parameters	Hazard Ratio	Lower 0.95	Upper 0.95	*p*-Value
CD3 S	1.000725	0.999777	1.001674	0.133869
CD4 S	0.998226	0.996616	0.999839	**0.031084**
CD8 S	0.998329	0.997033	0.999626	**0.011575**
CD20 S	1.00113	0.999907	1.002355	0.070211
CD68 S	1.000301	0.999701	1.0009	0.325558
CD163 S	1.000102	0.999669	1.000535	0.644591
iNOS S	1.000123	0.992674	1.007629	0.974216
DC-LAMP S	0.998948	0.994303	1.003614	0.657925
FOXP3 S	1.004488	1.000889	1.008101	**0.01449**
CD3 IT	1.01572	0.988186	1.04402	0.265969
CD4 IT	0.922045	0.846725	1.004065	0.061949
CD8 IT	1.023522	1.001187	1.046354	**0.038888**
CD20 IT	1.050171	0.64113	1.720179	0.84584
CD68 IT	0.985977	0.957906	1.01487	0.337894
CD163 IT	1.01417	1.002567	1.025907	**0.016547**
iNOS IT	0.997833	0.972125	1.02422	0.870592
DC-LAMP IT	0.997575	0.936183	1.062992	0.940266
FOXP3 IT	0.991568	0.953417	1.031245	0.672285
Sex	0.727182	0.435132	1.215248	0.224019
Age	0.982572	0.959449	1.006252	0.147893
Size	1.009228	0.98936	1.029495	0.365219
UICCIB	1.506231	0.330167	6.871471	0.596841
UICCIIA	2.930027	0.419885	20.4462	0.278136
UICCIIB	2.71031	0.656752	11.18501	0.168012
UICCIII	3.307966	0.700725	15.61617	0.130831
UICCIV	82.59885	10.07591	677.117	**3.92 × 10^−5^**
TLT	0.541932	0.286001	1.026886	0.060298
Anatomical-HEAD	0.749378	0.142995	3.927188	0.732818
Anatomical-TAIL	1.001193	0.178253	5.623408	0.99892
Budding grade	0.757975	0.513434	1.118988	0.163231
Budding number	1.057654	1.033902	1.081952	**1.32 × 10^−6^**
Model Performance	*p* value			
logtest	**7.47 × 10^−12^**			

Bolded values denote significant results.

## Data Availability

This study used publicly available datasets from The Cancer Genome Atlas available from (https://portal.gdc.cancer.gov/, accessed on 7 July 2020). All other data supporting the findings of this study can be found with the article or supplementary material.

## Supplementary Materials

The following are available online at https://www.mdpi.com/2072-6694/13/5/1090/s1, Figure S1: Study design, Figure S2: Tumor budding categories and number of tumor buds are associated with overall survival in PDAC, Figure S3: αSMA and Collagen histological scores in high grade versus low grade budding groups, Figure S4: Representative immunohistochemistry of immune cell profiles. Images from a selection of immunohistochemical stains of immune cells markers in High and Low Grade budding cases. The following markers were assessed for immune profiling. Pan-cytokeratin (brown chromogen) to stain tumor tissue and CD3, CD4, CD8 (red chromogen) representing lymphocyte subsets as well as iNOS, CD163 and CD68 (red chromogen) representing myeloid cell populations, Figure S5: Tertiary lymphoid tissues in High and Low Grade budding cases, Figure S6: Principal Component Analysis (PCA) shows distinct immune profiles in High versus Low Grade Budding cases, Table S1: Intra-tumoral (IT) immune profiles of High Grade versus Low Grade Budding cases, Table S2: Stromal (S) immune profiles of High Grade versus Low Grade Budding cases, Table S3: Distribution of EPIC-derived cell abundance values between high and low budding gene score patients from TCGA PAAD cohort.
