# Supplementary material for: Pancreatic Cancers with High Grade Tumor Budding Exhibit Hallmarks of Diminished Anti-Tumor Immunity
Source: Cancers (Basel). 2021 Mar 4;13(5):1090. doi: 10.3390/cancers13051090 (PMC7961597; doi:10.3390/cancers13051090)
Supplement: Supplementary file 1 [file cancers-13-01090-s001.pdf]

cancers

Supplementary Materials: Pancreatic Cancers with High Grade Tumor Budding Exhibit Hallmarks of Diminished Anti-tumor Immunity

Hassan Sadozai, Animesh Acharjee, Thomas Gruber, Beat Gloor and Eva Karamitopoulou

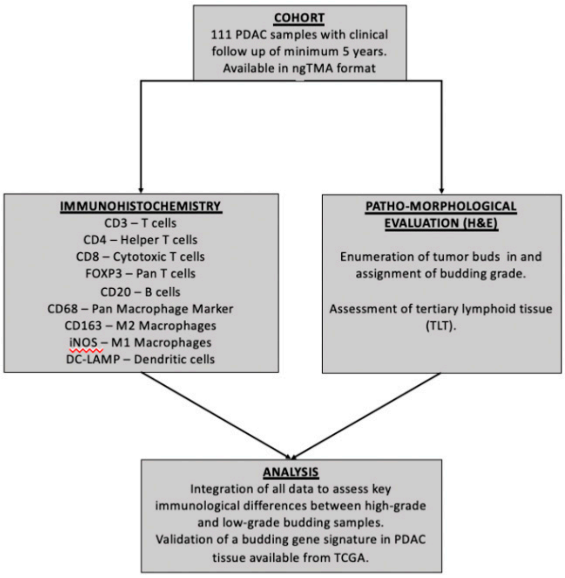

Figure S1. Study design.

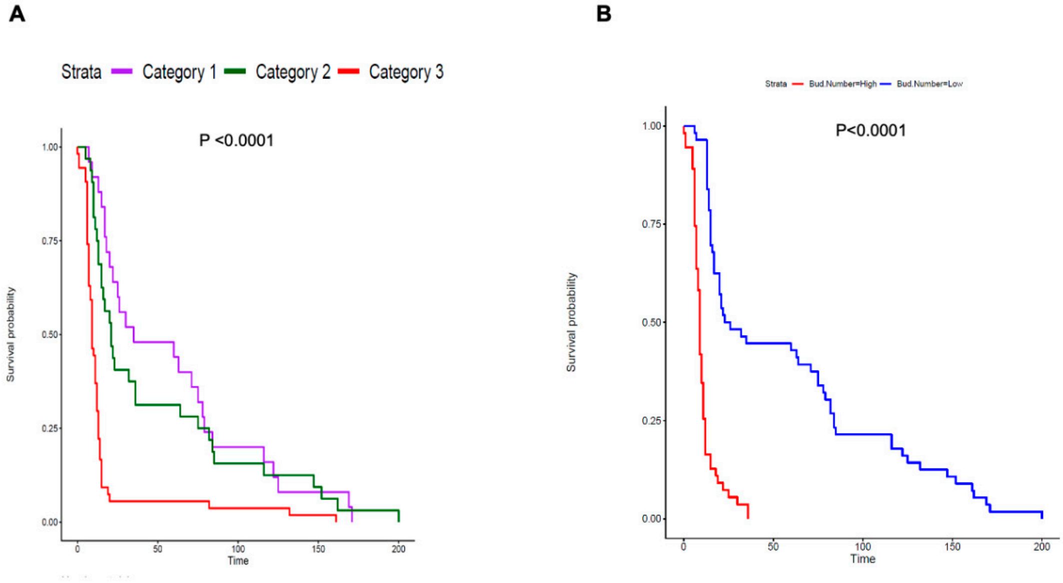

Figure S2. Tumor budding categories and number of tumor buds are associated with overall survival in PDAC. (**A**) Kaplan-Meier curves comparing OS of PDAC patients belonging to each of the 3 categories of tumor budding (Category 1,2 and 3). There were 25, 32 and 54 PDAC patients in

Category 1, 2 and 3 respectively. (**B**) Kaplan-Meier curves comparing OS of PDAC patients with high (red) or low (blue) numbers of tumor buds. Patients were assigned to high or low groups based a median dichotomization (*N*= 55 and N= 56 patients per group respectively). Statistical comparisons were performed using the log-rank test and shown in the KM plot.

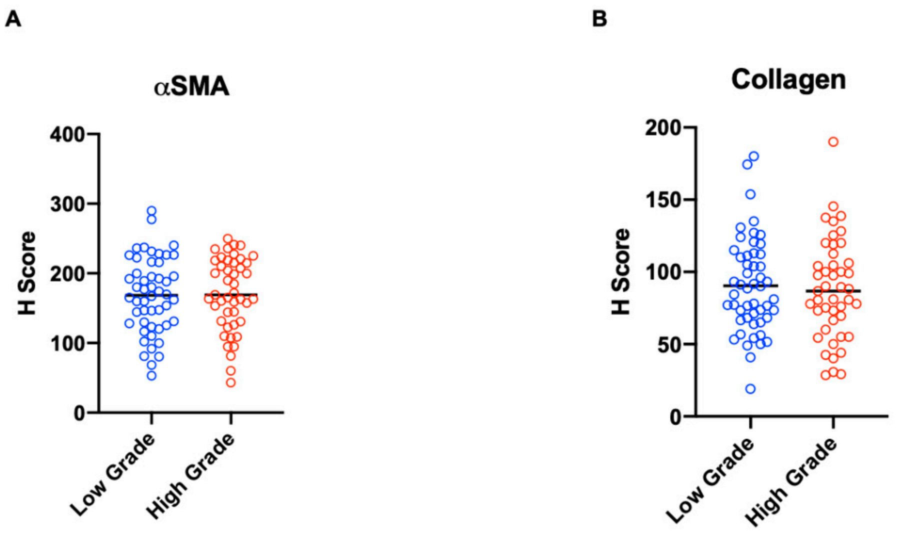

Figure S3. αSMA and Collagen histological scores in high grade versus low grade budding groups. (**A**) Scatterplots depicting H-Scores for αSMA staining (IHC) in HG vs. LG groups. (**B**) Scatterplots depicting H-Scores for Collagen staining (Masson's trichrome) in HG vs. LG cases. Mann-Whitney U test was used to determine differences between groups. Each symbol denotes an individual patient. The bars denote median values for each group.

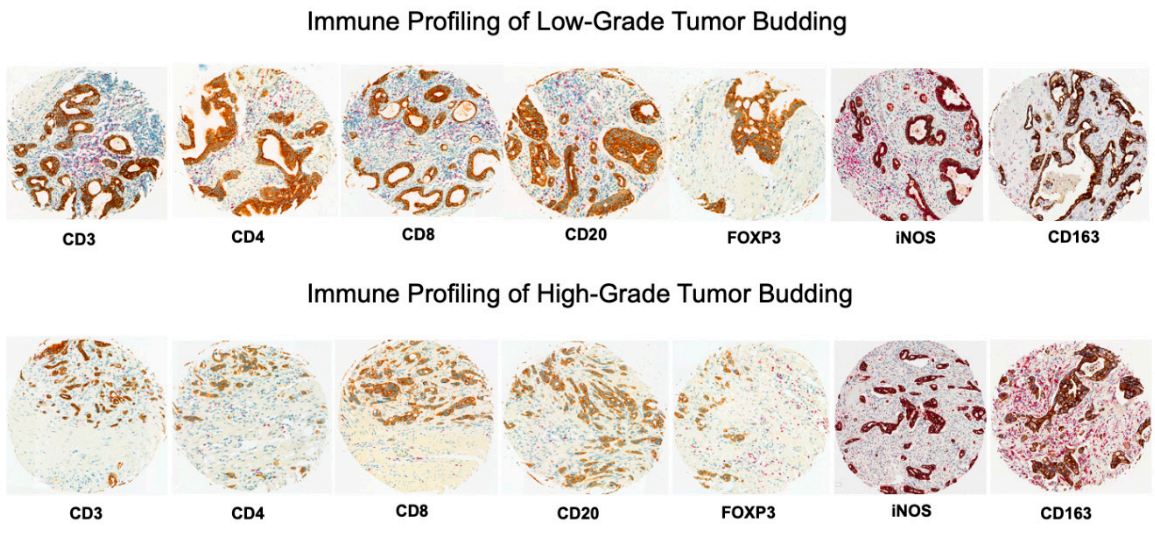

Figure S4. Representative immunohistochemistry of immune cell profiles. Images from a selection of immunohistochemical stains of immune cells markers in High and Low Grade budding cases. The following markers were assessed for immune profiling. Pan-cytokeratin (brown chromogen) to stain tumor tissue and CD3, CD4, CD8 (red chromogen) representing lymphocyte subsets as well as iNOS, CD163 and CD68 (red chromogen) representing myeloid cell populations.

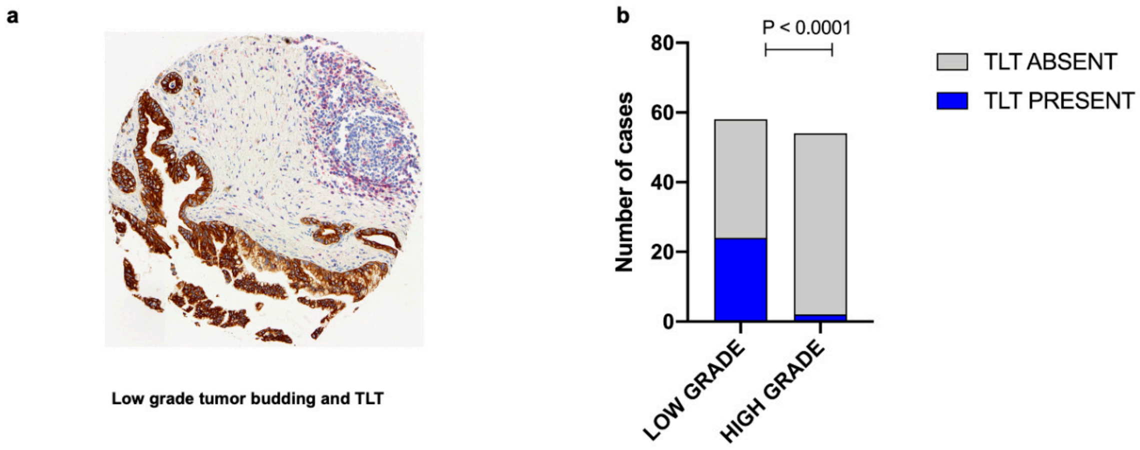

Figure S5. Tertiary lymphoid tissues in High and Low Grade budding cases**.** **(A)** Representative images showing PDAC cases with and without tertiary lymphoid tissue (TLT) (x200). CD3 (Red chromogen) and Pan-cytokeratin (Brown chromogen) are depicted. (**B**) Bar-graphs depicting the number of cases exhibiting presence or absence of tertiary lymphoid tissue (TLT) in High Grade (N = 54) and Low Grade (N = 57) patients. Statistical comparisons were performed using Fisher's exact test.

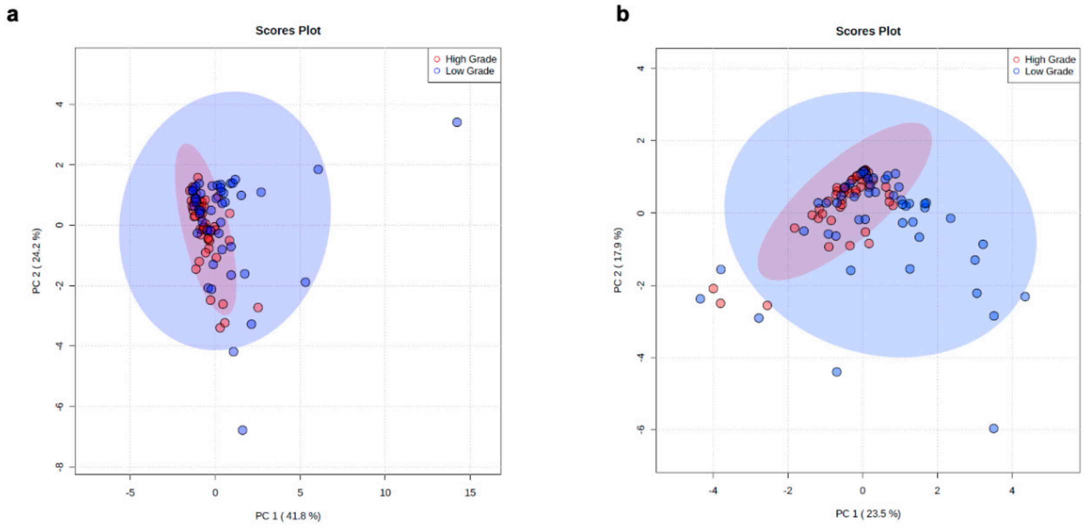

Figure S6. Principal Component Analysis (PCA) shows distinct immune profiles in High versus Low Grade Budding cases**.** PCA performed using (**A**) stromal and (**B**) intratumoral immune cell densities show clustering of High Grade (N = 54) and Low Grade (N = 57) budding PDAC patients. Each circle denotes an individual patient, high grade in red and low grade in blue. Two 95% confidence ellipses are drawn within each budding category. Low grade samples (blue) are spread more indicating patients are behaving differently from high grade budding (red). The percent variation is explained by first principal component (PC1, x-axis) and the second principal component -2 (PC2, y-axis).

Table S1. Intra-tumoral (IT) immune profiles of High Grade versus Low Grade Budding cases.

	High Grade		Low Grade		
**Immune Cell Type**	**Median**	**Range**	**Median**	**Range**	**p-Value**
**CD3/mm^2**	0.00	0.00–15.87	5.02	0.00–67.31	**0.0003**

**CD4/mm²**	0.00	0.00–4.78	0.00	0.00–42.55	**0.003**
**CD8/mm²**	0.00	0.00–36.08	4.61	0.00–131.90	**0.004**
**Foxp3/mm²**	0.00	0.00–28.89	0.00	0.00–82.34	0.25
**CD20/mm²**	0.00	0.00–1.53	0.00	0.00–3.53	0.36
**CD68/mm²**	3.90	0.00–132.50	0.68	0.00–89.73	0.17
**CD163/mm²**	17.73	0.00–165.50	5.56	0.00–142.40	**0.042**
**iNOS/mm²**	0.00	0.00–7.68	0.00	0.00–61.44	0.24
**DC-LAMP/mm²**	0.00	0.00–8.85	0.00	0.00–30.37	0.07

Bolded values denote significant results. Data are shown as median and range but were plotted in the main manuscript as mean + standard error.

Table S2. Stromal (S) immune profiles of High Grade versus Low Grade Budding cases.

	**High Grade**		**Low Grade**		
**Immune Cell Type**	**Median**	**Range**	**Median**	**Range**	**P value**
**CD3/mm²**	283.60	3.25–2647.00	566.90	83.21–3163.00	**0.0004**
**CD4/mm²**	20.50	0.00–308.70	55.27	0.00–4367.00	**0.0001**
**CD8/mm²**	264.80	0.00–1430.00	401.60	29.06–2635.00	**0.020**
**Foxp3/mm²**	39.49	0.00–531.90	20.74	0.00–528.50	**0.038**
**CD20/mm²**	15.53	0.00–178.50	49.97	0.00–5811.00	**<0.0001**
**CD68/mm²**	760.00	84.50–6730.00	566.10	169.00–4116.00	0.053
**CD163/mm²**	845.80	65.46–3497.00	552.90	65.50–6578.00	0.11
**iNOS/mm²**	6.39	0.00–63.14	15.80	0.00–481.50	**0.004**
**DC-LAMP/mm²**	11.55	0.00–126.00	31.91	0.00–617.00	**0.002**

Bolded values denote significant results. Data are shown as median and range but were plotted in the main manuscript as mean + standard error.

Table S3. Distribution of EPIC-derived cell abundance values between high and low budding gene score patients from TCGA PAAD cohort.

	**High Score**		**Low Score**		
**Immune Cell Type**	**Median**	**Range**	**Median**	**Range**	***P*-Value**
B cells	2.12×10^{-4}	3.42×10^{-9}–2.26×10^{-2}	4.45×10^{-3}	4.38×10^{-6}–1.54×10^{-1}	**0.0005**

Cancer Associated Fibroblasts	7.13×10^{-1}	7.57×10^{-2}–1.00	4.02×10^{-1}	4.12×10^{-3}–1.00	**0.0001**
CD4 T cells	6.55×10^{-2}	3.05×10^{-9}–2.09×10^{-1}	7.57×10^{-2}	9.98×10^{-8}–2.37×10^{-1}	**0.021**
CD8 T cells	5.05×10^{-6}	6.89×10^{-9}–4.39×10^{-2}	3.03×10^{-3}	1.49×10^{-8}–7.74×10^{-2}	**0.006**
Endothelial cells	7.03×10^{-2}	1.66×10^{-9}–1.72×10^{-1}	1.08×10^{-1}	1.98×10^{-7}–3.72×10^{-1}	**0.0004**
Macrophages	3.58×10^{-3}	1.68×10^{-9}–1.28×10^{-2}	5.79×10^{-3}	5.57×10^{-8}–5.60×10^{-2}	**0.004**
NK cells	6.19×10^{-8}	4.47×10^{-10}–8.86×10^{-4}	1.59×10^{-8}	6.41×10^{-11}–1.07×10^{-4}	**0.031**
Uncharacterized cells	1.47×10^{-1}	1.01×10^{-11}–7.62×10^{-1}	3.94×10^{-1}	5.28×10^{-10}–9.14×10^{-1}	**0.023**

Bolded values denote significant results. Data are shown as median and range but were plotted in the main manuscript as box and whisker plots.
